# Cascaded Spatial and Depth Attention UNet for Hippocampus Segmentation

**DOI:** 10.3390/jimaging11090311

**Published:** 2025-09-11

**Authors:** Zi-Zheng Wei, Bich-Thuy Vu, Maisam Abbas, Ran-Zan Wang

**Affiliations:** Department of Computer Science & Engineering, Yuan Ze University, Taoyuan 320, Taiwan; s1116036@mail.yzu.edu.tw (Z.-Z.W.); s1116054@mail.yzu.edu.tw (B.-T.V.); s1129105@mail.yzu.edu.tw (M.A.)

**Keywords:** hippocampus segmentation, U-Net, MRI images, image segmentation, deep learning

## Abstract

This study introduces a novel enhancement to the UNet architecture, termed Cascaded Spatial and Depth Attention U-Net (CSDA-UNet), tailored specifically for precise hippocampus segmentation in T1-weighted brain MRI scans. The proposed architecture integrates two key attention mechanisms: a Spatial Attention (SA) module, which refines spatial feature representations by producing attention maps from the deepest convolutional layer and modulating the matching object features; and an Inter-Slice Attention (ISA) module, which enhances volumetric uniformity by integrating related information from adjacent slices, thereby reinforcing the model’s capacity to capture inter-slice dependencies. The CSDA-UNet is assessed using hippocampal segmentation data derived from the Alzheimer’s Disease Neuroimaging Initiative (ADNI) and Decathlon, two benchmark studies widely employed in neuroimaging research. The proposed model outperforms state-of-the-art methods, achieving a Dice coefficient of 0.9512 and an IoU score of 0.9345 on ADNI and Dice scores of 0.9907/0.8963 (train/validation) and an IoU score of 0.9816/0.8132 (train/validation) on the Decathlon dataset across multiple quantitative metrics. These improvements underscore the efficacy of the proposed dual-attention framework in accurately explaining small, asymmetrical structures such as the hippocampus, while maintaining computational efficiency suitable for clinical deployment.

## 1. Introduction

The hippocampus, a quintessential neuroanatomical structure within the medial temporal lobe, represents one of the most extensively investigated regions in contemporary neuroscience due to its fundamental role in episodic memory consolidation, spatial cognition, and neuroplasticity [1]. This crescent-shaped structure, characterized by its intricate cytoarchitectural organization and distinct laminar arrangement, has emerged as a critical biomarker for numerous neurological and psychiatric pathologies, including Alzheimer’s disease, temporal lobe epilepsy, major depressive disorder, and post-traumatic stress disorder. The morphometric analysis of hippocampal volume, surface area, and shape characteristics has profound clinical implications for elucidating disease progression trajectories, therapeutic response monitoring, and prognostic stratification across diverse patient populations [2].

The advent of high-resolution magnetic resonance imaging (MRI) has revolutionized our ability to visualize and quantify hippocampal morphology with extraordinary precision [3]. However, the precise delineation of hippocampal boundaries remains a formidable computational challenge due to the structure’s complex three-dimensional geometry, substantial inter-individual anatomical variability, and the inherent ambiguity in defining precise anatomical boundaries adjacent to nearby structures such as the amygdala, parahippocampal gyrus, and ventricular system [4]. Traditional manual segmentation approaches, while considered the gold standard, are prohibitively time-consuming, subject to significant inter-rater variability, and fundamentally unsuitable for large-scale neuroimaging studies and clinical applications requiring rapid, automated analysis [5].

Contemporary automated segmentation methodologies have evolved from rudimentary atlas-based approaches to sophisticated machine learning paradigms, with deep learning architectures demonstrating remarkable efficacy in addressing the complexities inherent in neuroanatomical segmentation tasks [6]. The U-Net architecture, originally designed for biomedical image segmentation, has emerged as a foundational framework due to its elegant encoder–decoder structure with skip connections that preserve fine-grained spatial information while enabling hierarchical feature extraction [7]. However, conventional U-Net implementations often struggle with the nuanced spatial relationships and depth-dependent features that characterize hippocampal morphology, particularly in pathological conditions where alterations may manifest as subtle volumetric changes or irregular surface deformations [8].

The integration of attention mechanisms into deep learning networks has precipitated a paradigm shift in computer vision and medical image analysis, enabling networks to selectively focus on salient features while suppressing irrelevant information. Spatial attention mechanisms enable dynamic weighting of feature maps according to their spatial significance, thereby enhancing the network’s ability to discriminate between anatomically relevant structures and background noise [9]. Concurrently, depth attention mechanisms enable selective emphasis on features across different network layers, promoting integration of multi-scale information that is crucial for accurate boundary delineation in complex anatomical structures [9].

Recent advances in hippocampal segmentation have demonstrated the potential of hybrid architectures that combine multiple attention mechanisms to address the multifaceted challenges inherent in neuroanatomical segmentation [10]. However, existing approaches often implement attention mechanisms in isolation or employ simplistic combinations that fail to leverage the synergistic potential between spatial and depth attention mechanisms [11]. Furthermore, most current methodologies lack the architectural sophistication necessary to handle the cascaded processing requirements of optimal hippocampal segmentation, where coarse-to-fine refinement is essential for achieving clinically acceptable accuracy [12]. The cascaded processing paradigm represents a biologically inspired approach that mimics the hierarchical processing mechanisms observed in visual cortex, where information progresses through successive layers of increasing complexity and specialization [12]. In the context of hippocampal segmentation, cascaded architectures enable the progressive refinement of segmentation masks through multiple stages, with each stage focusing on progressively finer anatomical details. This approach is particularly advantageous for hippocampal segmentation, where the initial identification of gross anatomical boundaries must be followed by precise delineation of complex surface topology and the resolution of ambiguous boundaries [13].

The convergence of spatial attention, depth attention, and cascaded processing within a unified U-Net framework presents an unprecedented opportunity to address the longstanding challenges in automated hippocampal segmentation [14]. This integrated approach leverages the complementary strengths of each component: spatial attention for enhanced boundary localization, depth attention for optimal feature integration across network layers, and cascaded processing for progressive refinement of segmentation accuracy. This synergistic combination can achieve segmentation accuracy that approaches or exceeds manual segmentation standards while maintaining the computational efficiency necessary for clinical deployment [15].

Today’s clinical applications demand segmentation algorithms that not only achieve high accuracy but also demonstrate robust performance across diverse imaging protocols, scanner manufacturers, and patient populations. The heterogeneity of clinical MRI data, including variations in field strength, sequence parameters, and image quality, presents additional challenges that must be addressed through sophisticated architectural design and comprehensive training strategies [16]. Moreover, the increasing emphasis on subfield-specific analysis of hippocampal subregions, including CA1, CA2, CA3, the dentate gyrus, and the subicular complex, necessitates segmentation algorithms capable of resolving fine anatomical distinctions with sub-millimeter precision [17]. The clinical significance of accurate hippocampal segmentation extends beyond volumetric quantification to include shape analysis, surface-based morphometry, and longitudinal change detection. Advanced segmentation algorithms must therefore provide not only accurate boundary delineation but also geometrically consistent representations that facilitate downstream analysis. The integration of attention mechanisms within cascaded architectures offers the potential to meet these demanding requirements while maintaining computational tractability for large-scale studies [18].

Hippocampus segmentation plays a crucial clinical role in the diagnosis and monitoring of neurodegenerative diseases, particularly Alzheimer’s disease and mild cognitive impairment. The hippocampus is one of the first brain regions to exhibit atrophy in Alzheimer’s disease progression, making accurate segmentation essential for early detection and therapeutic monitoring [19]. Quantitative hippocampal volume measurements derived from MRI segmentation enable clinicians to track disease progression, evaluate treatment efficacy, and predict cognitive decline with high precision [3]. Furthermore, automated hippocampus segmentation facilitates large-scale population studies and reduces inter-observer variability associated with manual delineation, providing reliable biomarkers for clinical decision-making. Recent studies have demonstrated that deep learning-based segmentation approaches achieve clinical-grade accuracy, making them valuable tools for routine clinical practice and longitudinal patient monitoring [14].

This study presents a novel Cascaded Spatial and Depth Attention UNet architecture specifically designed to address the multifaceted challenges of hippocampal segmentation. The proposed framework incorporates sophisticated attention mechanisms within a cascaded processing paradigm, enabling the progressive refinement of segmentation masks through multiple stages of increasing specificity [20]. The spatial attention component enables the dynamic weighting of feature maps based on their anatomical relevance, while the depth attention mechanism optimizes feature integration across network layers. The cascaded architecture enables hierarchical processing of imaging data, from coarse anatomical localization to fine-grained boundary refinement, thereby achieving segmentation accuracy that surpasses existing state-of-the-art methodologies [20].

The fundamental hypothesis underlying this research posits that synergistically integrating spatial attention, depth attention, and cascaded processing within a unified U-Net framework will yield superior hippocampal segmentation performance compared to conventional approaches. This hypothesis is grounded in the recognition that hippocampal segmentation requires simultaneous consideration of spatial context, multi-scale feature integration, and hierarchical processing, capabilities that are optimally addressed through the proposed architectural innovation. The subsequent sections of this manuscript provide comprehensive validation of this hypothesis through extensive experimental evaluation on diverse datasets and rigorous comparison with established benchmarks.

## 2. Related Works

### 2.1. U-Net Network

The U-Net architecture, originally proposed by Ronneberger et al. [7], has fundamentally transformed biomedical image segmentation through its innovative encoder–decoder architecture with skip connections. This seminal contribution introduced a revolutionary approach to semantic segmentation that addresses the inherent trade-off between spatial localization precision and contextual understanding, a challenge that had persistently affected traditional convolutional neural network architectures in medical imaging applications.

The architectural elegance of U-Net lies in its symmetrical structure, where the contracting path (encoder) systematically reduces spatial dimensions while increasing feature map depth, enabling the extraction of increasingly abstract and semantically rich features. Conversely, the expansive path (decoder) progressively restores spatial resolution through transposed convolutions while integrating high-level contextual information. The distinctive skip connections establish direct pathways between corresponding encoder and decoder layers, facilitating the preservation of fine-grained spatial details that would otherwise be lost during the dimensionality reduction process. Contemporary adaptations of the U-Net framework have demonstrated remarkable versatility across diverse medical imaging modalities and anatomical structures. Singh et al. [17] introduced a multi-scale U-Net variant that incorporates dilated convolutions at multiple scales, achieving superior performance in cardiac MRI segmentation by capturing both local anatomical details and global contextual relationships. Similarly, Sharma et al. [18] proposed a residual U-Net architecture that integrates residual learning principles, demonstrating enhanced gradient flow and improved convergence characteristics for brain tumor segmentation tasks.

The application of U-Net to hippocampal segmentation has revealed both its strengths and inherent limitations. While the architecture excels at capturing gross anatomical boundaries, it often struggles with the subtle morphological variations and complex geometric topology that characterizes hippocampal structure. Sohail et al. [21] demonstrated that modified U-Net implementations frequently produce segmentation masks with irregular boundaries and insufficient accuracy in disambiguating hippocampal subfields, particularly in pathological conditions where structural integrity may be compromised. Recent innovations have focused on addressing these limitations through architectural modifications and refined training strategies. The introduction of dense skip connections by Wang et al. [20] enhanced feature propagation efficiency, while the incorporation of squeeze-and-excitation blocks by Kunjumon et al. [22] improved channel-wise feature recalibration. However, these enhancements remain insufficient for addressing the multifaceted challenges inherent in hippocampal segmentation, necessitating more sophisticated attention mechanisms and processing paradigms.

### 2.2. Spatial Attention U-Net Network (SA-UNet)

The integration of spatial attention mechanisms into U-Net architectures represents a significant advancement in addressing the spatial heterogeneity and anatomical complexity inherent in neuroanatomical segmentation tasks. Spatial attention mechanisms enable neural networks to dynamically modulate feature map activations according to their spatial significance, effectively implementing a learned weighting scheme that emphasizes anatomically relevant regions while suppressing background noise.

The foundational work of Oktay et al. [23] introduced attention gates into U-Net architectures, demonstrating that spatial attention could significantly improve segmentation accuracy by enabling the network to focus on salient anatomical features. This pioneering approach employed attention coefficients computed through a gating mechanism that combined features from both encoder and decoder pathways, creating a spatially aware feature selection process that enhanced boundary localization accuracy. Contemporary SA-UNet implementations have evolved beyond simple attention gating to incorporate sophisticated spatial attention modules that leverage multi-scale contextual information. Li et al. [24] proposed a dual multi-scale spatial attention U-Net mechanism that simultaneously processes features at multiple resolutions, enabling the capture of both fine-grained anatomical details and broader contextual relationships. Their approach demonstrated superior performance in medical image segmentation, including in hippocampal segmentation by effectively handling the multi-scale nature of hippocampal morphology, from gross anatomical boundaries to intricate subfield delineations. Wang and Yu [25] introduced a convolutional attention mechanism that employs learnable spatial filters to generate attention maps, moving beyond simple pooling-based approaches to create more sophisticated spatial weighting schemes. Their approach demonstrated efficacy in handling the irregular geometry of hippocampal structures, where traditional attention mechanisms often fail to capture the complex spatial relationships between different anatomical regions.

Advanced SA-UNet variants have incorporated deformable convolutions within attention modules, enabling adaptive spatial sampling that accommodates the natural anatomical variations observed in hippocampal morphology. The research by Chen et al. [26] demonstrated that dual multilevel constrained attention GAN could significantly improve segmentation accuracy in cases of hippocampal sclerosis, where traditional rigid attention mechanisms proved inadequate for capturing the altered anatomical topology.

### 2.3. Spatial Attention-Based Densely Connected U-Net (SAU-Net)

The fusion of spatial attention mechanisms with densely connected architectures represents a sophisticated approach to addressing feature propagation challenges inherent in deep segmentation networks. SAU-Net architectures leverage the complementary strengths of dense connectivity and spatial attention to create networks that simultaneously excel at feature reuse and spatial localization, addressing critical limitations observed in conventional U-Net implementations. Dense connectivity, originally introduced in DenseNet architectures by Huang et al. [27], enables each layer to receive feature inputs from all preceding layers, creating a rich feature propagation network that mitigates gradient vanishing problems and promotes feature reuse. The integration of this principle into U-Net architectures has demonstrated substantial improvements in segmentation performance, particularly for tasks requiring fine-grained anatomical discrimination.

The pioneering work of Chen et al. [28] established the foundation for SAU-Net architectures by demonstrating that densely connected U-Net variants could achieve superior performance in medical image segmentation tasks. Their approach incorporated dense blocks within both encoder and decoder pathways, enabling more effective feature propagation and reducing the number of trainable parameters through feature reuse. The advancement of automated hippocampus segmentation methodologies has seen substantial progress through the development of sophisticated deep learning architectures and hybrid frameworks that address the multifaceted challenges inherent in neuroanatomical delineation. Contemporary research demonstrates a convergence toward integrating multiple attention mechanisms, advanced preprocessing techniques, and specialized network architectures to achieve superior segmentation accuracy.

Sanjay and Swarnalatha [29] introduced a comprehensive framework employing the Kullback–Leibler Within-layer Regularized U-Net (KLW-RU-Net) combined with Type-1 Fuzzy Logic (T1FL) for dominant hippocampus segmentation, achieving enhanced AD subtype classification through brain atrophy analysis and incorporating Knowledge Partitioned Clustering (KPC) for tissue segmentation alongside Fisher-Kolmogorov (FK) model-based spatiotemporal evaluation. This work demonstrated the efficacy of integrating multiple analytical approaches within a unified framework for improved diagnostic accuracy.

Ding et al. [30] developed the Parallel Cascaded Feature Reconstruction Network (PCFR-Net), a lightweight hybrid segmentation architecture that synergistically combines global self-attention mechanisms with local convolution operations, incorporating a feature reconstruction module and a multibranch asymmetric residual attention module to achieve remarkable performance with DSC of 92.74% and IoU of 86.5% on the Medical Segmentation Decathlon dataset, and DSC of 93.86% and IoU of 89.29% on the ADNI dataset.

The integration of transformer architectures with traditional U-Net frameworks has been further explored by Sun et al. [16], who proposed DA-TransUNet, incorporating spatial and channel dual attention mechanisms with transformer U-Net architecture specifically tailored for medical image segmentation’s high-detail requirements, demonstrating consistent superiority across five datasets through optimized intermittent channels and strategic dual attention deployment in skip-connection.

Khan et al. [31] addressed the challenges of limited dataset variability through a resource-efficient 3D U-Net framework enhanced with 3D Contrast Limited Adaptive Histogram Equalization (CLAHE) and Selective Coefficient-Enhanced 3D Wavelet Transform (SCE-3DWT) preprocessing pipeline, achieving notable performance with a Dice coefficient of 0.8838 and a Jaccard Index of 0.7920 on the EADC-ADNI HarP dataset while maintaining computational efficiency through lightweight architecture design. These collective contributions underscore the evolution toward multi-modal attention mechanisms, sophisticated preprocessing strategies, and hybrid architectural designs that collectively advance the state-of-the-art in automated hippocampus segmentation for clinical applications.

Rundo & Militello [32] emphasize the importance of balancing handcrafted and deep-learned features in radiomics, highlighting the role of explainable AI in enhancing the interpretability of biomarkers like hippocampal volume and shape for Alzheimer’s disease diagnosis. They note that dataset size and diversity are critical to avoid overfitting, advocating for multi-institutional federated learning to improve model robustness. Similarly, Bacon et al. [33] provide a systematic review of AI-driven neuroimaging, showing that deep learning excels in disease classification (58.7% of studies) and lesion segmentation (28.9%), significantly improving diagnostic precision for neurological disorders.

## 3. Proposed Method

### 3.1. Network Architecture

**Figure 1:**
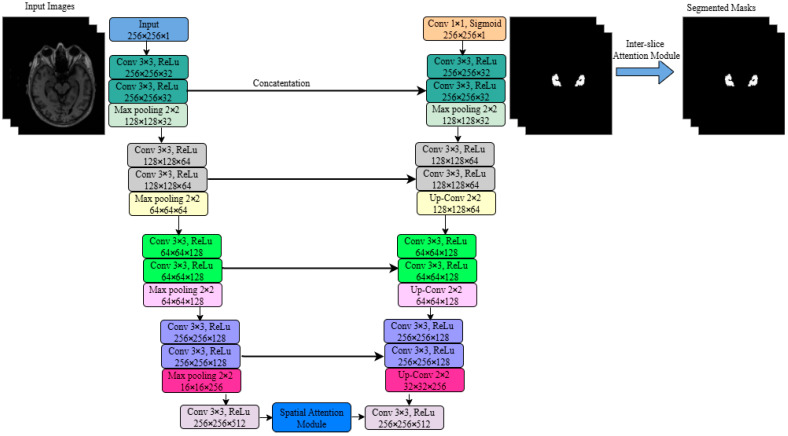
Model architecture diagram of the proposed CSDA-UNet method.

Figure 1 illustrates the proposed CSDA-UNet architecture. Its structure follows the U-Net design while adding a Spatial Attention module (SA module) and an Inter-Slice Attention module (ISA module). The input to CSDA-UNet is an MRI image with a size of 256 × 256 × 1.

The encoding path consists of five layers of convolution and pooling operations. In each layer, the input feature map undergoes a dual 3 × 3 convolution operation, with a ReLU activation function applied after each dual convolution layer. Finally, a 2 × 2 max pooling layer reduces the size of the feature map by half. The output sizes for each layer are as follows: the first layer outputs 256 × 256 × 32, becoming 128 × 128 × 32 after pooling; the second layer outputs 128 × 128 × 64, becoming 64 × 64 × 64 after pooling; the third layer outputs 64 × 64 × 128, becoming 32 × 32 × 128 after pooling; the fourth layer outputs 32 × 32 × 256, becoming 16 × 16 × 256 after pooling; and the fifth layer outputs 16 × 16 × 512, which is further optimized by the SA module.

The decoding path recovers the spatial dimensions of the feature maps through successive deconvolution and convolution operations. The first layer upsamples the feature map to 32 × 32 × 256 using a 2 × 2 upsampling layer, followed by dual 3 × 3 convolutions, and establishes a skip connection with the feature map from the fourth layer of the encoding path. The second layer upsamples to 64 × 64 × 128, performs dual 3 × 3 convolutions, and connects with the feature map from the third layer of the encoding path. The third layer upsamples to 128 × 128 × 64, performs dual 3 × 3 convolutions, and connects with the feature map from the second layer of the encoding path. The fourth layer upsamples to 256 × 256 × 32, performs dual 3 × 3 convolutions, and connects with the feature map from the first layer of the encoding path. Finally, a 1 × 1 convolution layer followed by a Sigmoid activation function generates a segmentation map of size 256 × 256 × 1. After the decoding path, the ISA module further processes the feature map to improve segmentation accuracy.

### 3.2. Spatial Attention (SA) Module

**Figure 2:**
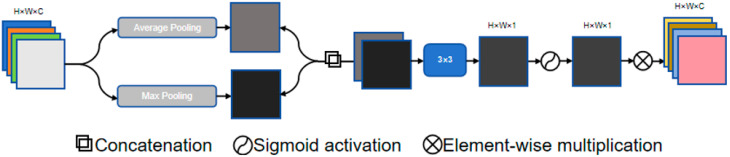
Schematic of the Spatial Attention (SA) module in CSDA-UNet, which highlights informative spatial regions and suppresses irrelevant ones to capture fine hippocampal details in MRI scans.

The SA module inserted at the bottom of the network generates a spatial attention map that is overlaid on the convolutional layers, as shown in Figure 2. This module first applies max pooling and average pooling to the input feature map to generate two feature descriptor maps. These descriptor maps are then concatenated and processed through convolution operations. The SA module uses a set of convolution kernel sizes, including 1 × 1, 3 × 3, 5 × 5, and 7 × 7, ultimately producing an attention map through a Sigmoid activation function. This attention map further emphasizes the features captured by the convolutional layers, thereby improving segmentation accuracy.

### 3.3. Inter-Slice Attention (ISA) Module

**Figure 3:**
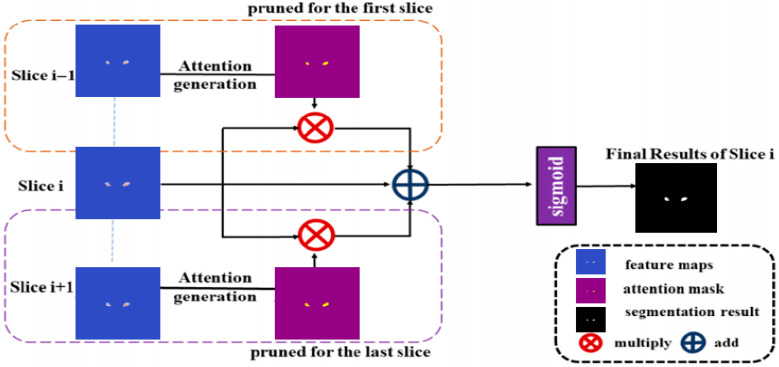
Schematic of the Inter-Slice Attention (ISA) module in CSDA-UNet, which exploits contextual cues across adjacent MRI slices to improve segmentation continuity and consistency.

The ISA module is integrated at the final stage of the decoding path to enhance cross-slice contextual awareness in 3D medical image segmentation. As illustrated in Figure 3, it leverages spatial dependencies between adjacent slices by generating attention maps from neighboring slice feature maps (Slice i − 1 and Slice i + 1) using a lightweight attention mechanism. These attention masks are then applied via element-wise multiplication to the current slice’s feature map (Slice i), effectively weighting relevant spatial regions. The refined features are fused through element-wise addition and passed through a 1 × 1 convolution followed by a Sigmoid activation to produce the final 256 × 256 × 1 segmentation output.

### 3.4. Images Preprocessing and Augmentation

**Figure 4:**
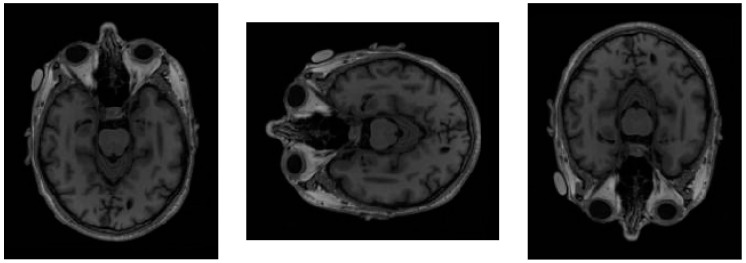
A sample image from the test dataset (**left**) alongside two augmentation result images: horizontal flipping (**middle**) and vertical flipping (**right**).

To enhance the model’s ability to recognize image features and to mitigate overfitting, several regularization strategies were employed during model training. Data augmentation techniques, including random horizontal and vertical flips and image enhancement, were applied to increase dataset diversity. Additionally, early stopping based on validation loss and careful learning rate scheduling were used to prevent overfitting. This approach increases the diversity of the training data without altering the inherent details and features of the images. Horizontal flipping simulates the left-right differences that may occur in anatomical structures among different individuals, while vertical flipping helps the model learn the impact of vertical position changes on pathological tissue features. In addition, image enhancement techniques were applied, including Contrast Limited Adaptive Histogram Equalization (CLAHE) to improve local contrast and Gaussian filtering to suppress noise while preserving structural boundaries. Figure 4 presents a sample image from the dataset alongside two types of random variations, one using horizontal flipping and the other using vertical flipping.

After the training is completed, a confusion matrix is used to calculate evaluation metrics for assessing model performance. The relevant metrics are defined in Table 1. These metrics represent the relationship between the model’s predicted results and the actual values in classification problems. Based on the confusion matrix, we can calculate evaluation metrics including Accuracy, Precision, Recall, F1 score, Dice coefficient, and the Intersection Over Union (IOU).

The accuracy formula is provided in Equation (1). This formula describes the ratio of the number of instances correctly predicted by the model to the total number of samples, serving as a fundamental metric for evaluating the overall performance of the model.

(1) $$Accuracy=\frac{TP+TN}{TP+FP+TN+FN}.$$

Equation (2) provides the formula for precision. This metric focuses on assessing the proportion of actual positives among the samples classified as positive by the model, reflecting the accuracy of the model’s positive determinations.

(2) $$Precision=\frac{TP}{TP+FP}.$$

Equation (3) provides the formula for recall, also known as the true positive rate or sensitivity. Recall measures the proportion of actual positive samples that are correctly identified by the model and is an important metric for evaluating the model’s ability to recognize positive samples. In contexts such as clinical diagnosis, a high recall indicates a lower rate of missed diagnoses.

(3) $$Recall=\frac{TP}{TP+FN}$$

The Dice coefficient is defined in Equation (4). The Dice coefficient is the ratio of twice the number of true positives to the sum of predicted positives and actual positives, and it is commonly used in the field of medical imaging.

(4) $$Dice\;coefficient=\frac{2\times TP}{2\times TP+FP+FN}.$$

Equation (5) provides the formula for Intersection Over Union (IOU), also known as the Jaccard Index. This metric measures the ratio of the overlapping area between the predicted positives and the actual positives to their union area.

(5) $$IOU=\frac{TP}{TP+FP+FN}$$

To ensure robust evaluation of the proposed CSDA-UNet and baseline models (U-Net, U-Net + SA, U-Net + ISA), we employed rigorous statistical methods to validate performance differences. Paired t-tests were conducted to compare the Dice coefficient and Intersection Over Union (IoU) scores between CSDA-UNet and each baseline model across the ADNI and Decathlon datasets. The null hypothesis assumes no significant difference in performance metrics between models, with a significance level of α = 0.05. Additionally, we implemented 5-fold cross-validation to assess model generalizability and reduce overfitting risks, particularly given the limited size of the ADNI dataset. Confidence intervals (95%) were calculated for key metrics (Dice, IoU) to quantify the precision of the reported results. These statistical analyses ensure that the performance improvements of CSDA-UNet are statistically significant and reliable for clinical applications.

### 3.5. Experimental Environment and Dataset

The experimental framework for this study was implemented using Python 3.8 and the PyTorch 2.0.1 deep learning library, executed within a CUDA 11.7-enabled environment to leverage GPU acceleration. The hardware and software configurations utilized for model development and training are summarized in Table 2.

**Figure 5:**
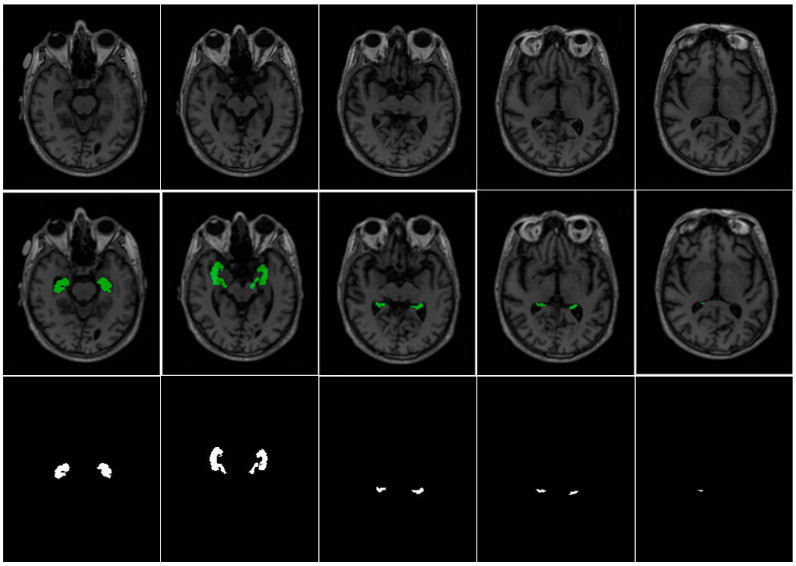
Axial MRI slices (**top**), mask overlays (**middle**), and ground truth (**bottom**) for the hippocampal gyrus at slices 71st, 76th, 81st, 86th, and 91st.

For empirical validation, we employed two hippocampus segmentation datasets: the Alzheimer’s Disease Neuroimaging Initiative (ADNI) dataset [34] and the Decathlon Hippocampus dataset [35]. Both datasets contain T1-weighted MRI scans and are publicly accessible resources for advancing Alzheimer’s Disease understanding and early diagnosis. The ADNI dataset comprises cross-sectional T1-weighted MRI scans from 135 subjects, with each subject contributing 189 axial slices (30 slices containing visible hippocampal structures on average). For model training and evaluation, data from 100 subjects (18,900 images) were allocated to the training set, while the remaining 35 subjects (6615 images) formed the test set. The Decathlon Hippocampus dataset includes T1-weighted MRI scans from 390 patients, with 30–34 slices per patient (9–10 slices with masks). The dataset was split into 260 patients (9270 images) for training and 130 patients (4499 images) for testing. Table 3 summarizes the ADNI and Decathlon datasets used for hippocampus segmentation in our experiments. A representative visualization of hippocampal ground truth annotations overlaid on corresponding MRI slices from the ADNI dataset is presented in Figure 5. This rigorously annotated dataset provides a robust foundation for benchmarking hippocampus segmentation models, particularly in the context of neurodegenerative disease assessment and clinical deployment.

In this study, the Adam optimizer is used, and the loss function is binary cross-entropy loss. Among the model parameters, the learning rate is set to 0.001, the batch size is 4, and the total number of training epochs is 200. Images are padded to a size of 256 × 256 through zero-padding before being input into the model and are cropped back to the original size during output to avoid image distortion.

## 4. Experimental Results

### 4.1. Ablation Study

The CSDA-UNet comprises 7.76 M parameters with standard GPU memory requirements, demonstrating efficient model complexity. On the Decathlon dataset, the model trained for 200 epochs in 14.6 h, with comparative training times on the ADNI dataset showing CSDA-UNet requires 3 h 45 min, representing reasonable computational overhead relative to baseline architectures (U-Net: 2 h 33 min, U-Net + SA: 2 h 50 min, U-Net + ISA: 1 h 8 min). Inference performance demonstrates clinical feasibility with approximately 0.38 s per slice processing time, confirming CSDA-UNet’s practical viability for real-time hippocampus segmentation in clinical deployment while maintaining superior segmentation accuracy.

After training with various configurations of convolutional blocks in the SA module, the experiments showed that using a 3 × 3 convolutional block allows the SA module to perform better in this segmentation task. The evaluation metrics of the training results after adjusting the size of the convolutional kernels in the SA module are shown in Table 4.

**Figure 6:**
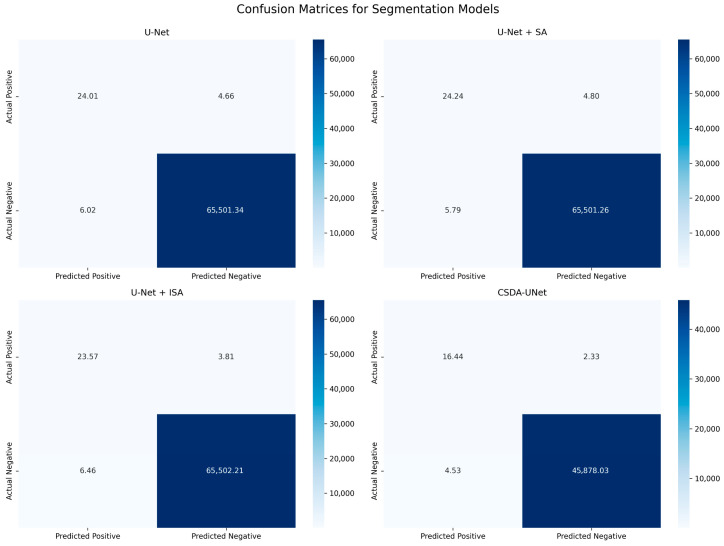
Confusion Matrices for Segmentation Models.

The ablation study results, presented in Table 5 and Table 6, highlight the performance improvements achieved through various enhancements to the baseline U-Net architecture for hippocampal MRI image segmentation. Specifically, the incorporation of preprocessing techniques along with the Spatial Attention (SA) and Inter-Slice Attention (ISA) modules leads to consistently higher evaluation metrics. The confusion matrix analysis shown in Figure 6 illustrates the performance differences across the evaluated models. The baseline U-Net achieves high true positives and true negatives but is slightly affected by false predictions, impacting its precision and recall. Adding a Spatial Attention (SA) module improves recall, while the ISA variant enhances precision by reducing false positives. The U-Net-CSDA further improves performance, achieving the highest overall metrics, precision (0.9644), F1 score (0.9529), Dice coefficient (0.9512), and a reduced number of false predictions. These results confirm its superior segmentation accuracy and robustness compared to previous models.

**Figure 7:**
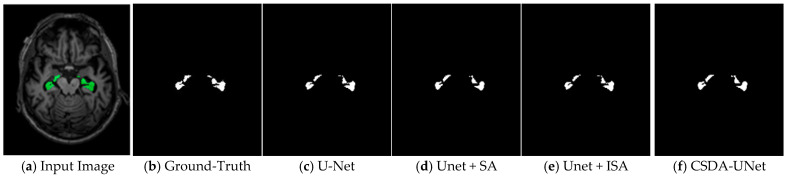
An example of hippocampus segmentation result. (**a**–**f**) illustrate, respectively: the original subject slice with mask overlay, the ground truth segmentation, the segmentation produced by the U-Net model, the U-Net with Spatial Attention (SA), the U-Net with Inter-Slice Attention (ISA), and the proposed CSDA-UNet segmentation output.

These improvements are further visualized in Figure 7. A comparison between configurations Figure 7c,d, as well as Figure 7e,f, demonstrates that adding the SA module significantly enhances the model’s ability to accurately localize the target region. Furthermore, comparing Figure 7c,e and Figure 7d,f indicates that while the ISA module contributes moderately to the segmentation of the hippocampal region, it still offers incremental gains in overall model performance.

Table 7 summarizes the Dice coefficient and IoU metrics for the evaluated models, focusing on statistical validation. CSDA-UNet demonstrates significant improvements over baselines on the ADNI dataset, with *p*-values indicating robust performance gains. The Decathlon dataset results highlight CSDA-UNet’s high accuracy and generalizability across train and validation sets.

### 4.2. Result Analysis

The comparison with related research papers is shown in Table 8, which contrasts the proposed CSDA-UNet method with methods presented in previous studies on the same hippocampal segmentation task. We compared our approach with the methods proposed by several scholars and used the Dice coefficient and accuracy as evaluation metrics.

**Figure 8:**
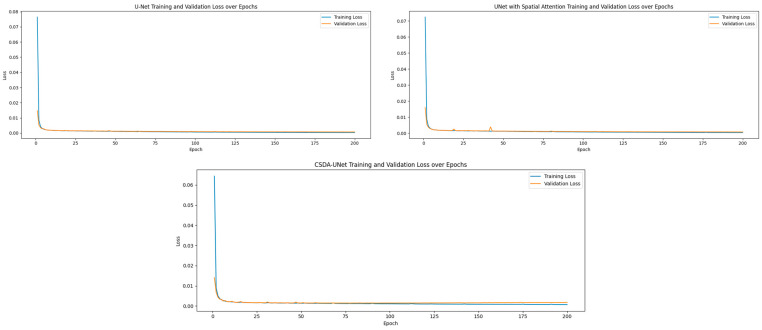
Training and validation loss over epochs for UNet (**top-left**), UNet + SA (**top-right**) and CSDA-UNet (**bottom**).

**Figure 9:**
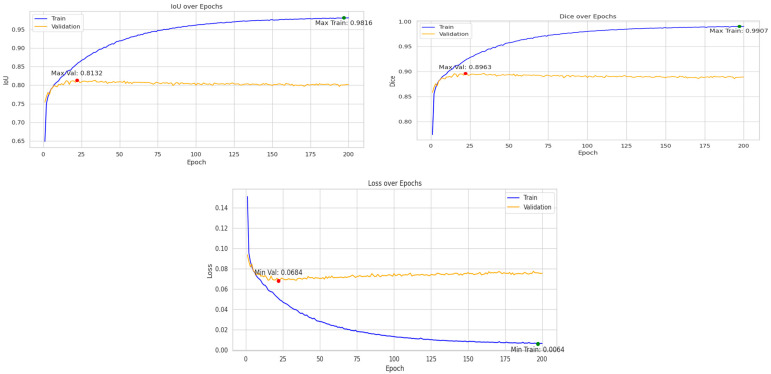
Training and validation metrics for the CSDA-UNet model on the Decathlon dataset over 200 epochs: IoU (**top left**), Dice score (**top right**), loss (**bottom**).

As demonstrated in Table 8, our CSDA-UNet achieves superior performance across both datasets. On the ADNI dataset, our method obtains a Dice coefficient of 0.9512 and accuracy of 0.98, outperforming Das et al.’s Modified 3D-UNet (0.9343 Dice, 0.90 accuracy) and Nisha et al.’s SSGAN (0.85 Dice, 0.95 accuracy). On the Decathlon dataset, our CSDA-UNet achieves 0.9907/0.8963 Dice coefficient (train/validation) and 0.9952/0.9832 accuracy (train/validation), surpassing recent transformer-based approaches, including Lei Yang et al.’s Morphological ViT (0.90–0.886 Dice) and Xiao Zhiyong et al.’s Swin-UNETR (0.878 Dice). Figure 8 illustrates a comparative analysis of the training and validation loss curves for U-Net, U-Net with Spatial Attention, and CSDA-U-Net on the ADNI dataset. Subsequently, Figure 9 presents the learning curves for the Dice coefficient, IoU, and loss on the Decathlon dataset.

**Figure 10:**
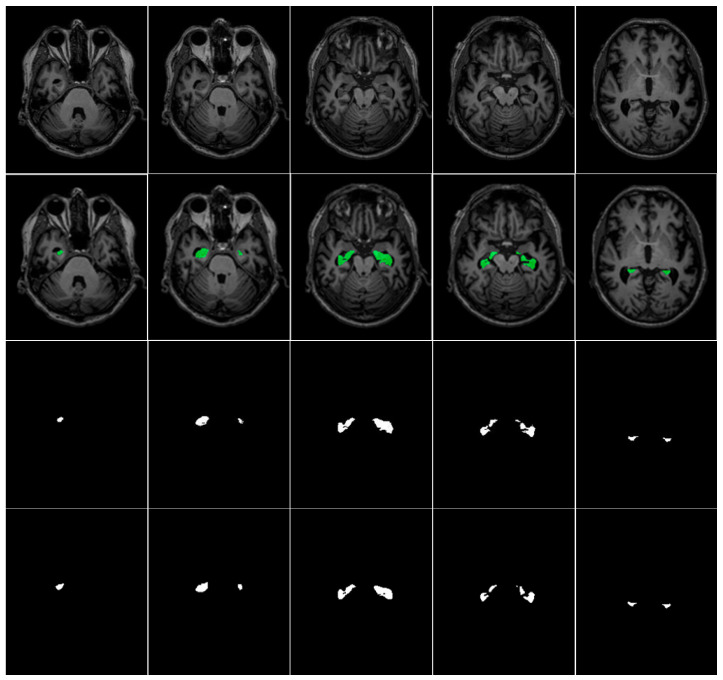
MRI images (**1st row**), masks overlays (**2nd row**), ground truth (**3rd row**), and CSDA U-Net model segmented regions (**4th row**) for ADNI Dataset.

**Figure 11:**
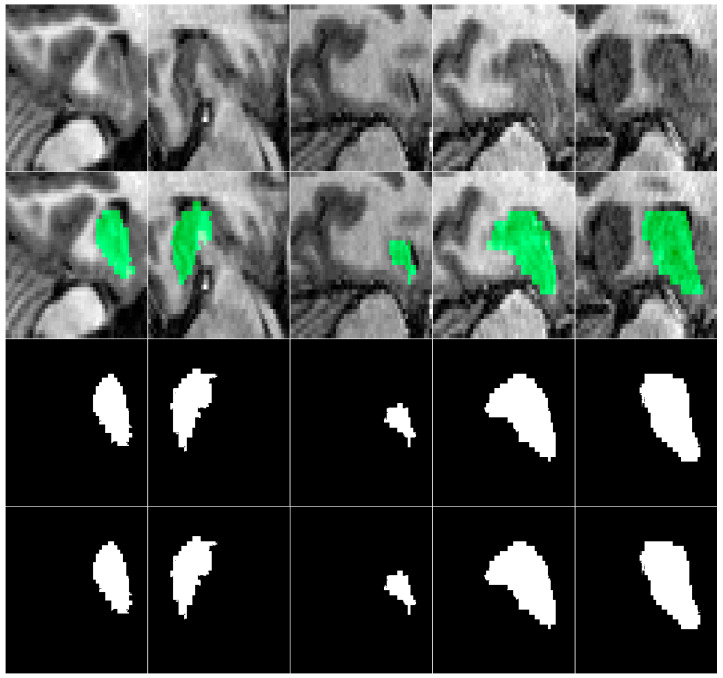
MRI images (**1st row**), masks overlays (**2nd row**), ground truth (**3rd row**), and CSDA U-Net model segmented regions (**4th row**) for Decathlon Dataset.

This indicates that CSDA-UNet, by incorporating multiple spatial attention modules and inter-slice attention modules, can better enhance the model’s ability to extract spatial features, thereby improving segmentation performance and stability. The slightly lower recall of CSDA-UNet arises from its focus on reducing false positives via channel–spatial dual attention, which improves precision but may miss some boundary regions, especially given the small size of hippocampal areas relative to the background. An illustration of the segmented regions after model training is shown in Figure 10 for the ADNI dataset and Figure 11 for the Decathlon dataset, demonstrating a high capability for hippocampal segmentation. CSDA-UNet showed only a slight increase in inference time over U-Net, U-Net + SA, and U-Net + ISA, while maintaining similar batch throughput. This minor cost is justified by its significant accuracy and precision gains, confirming its clinical practicality.

In summary, the experimental results indicate that CSDA-UNet performs exceptionally well in the hippocampal segmentation task, providing higher segmentation accuracy and robustness. This suggests that this method can offer strong support for early diagnosis and subsequent monitoring of various neurogenerative diseases in future research.

## 5. Conclusions

This research applies a UNet-based deep learning architecture that is augmented with a Spatial Attention (SA) module as well as an Inter-Slice Attention (ISA) module in order to achieve very precise segmentation of the hippocampal region in MRI images. By applying the proposed technique on human brain MRI scans sourced from the Alzheimer’s Disease Neuroimaging Initiative (ADNI) and Decathlon databases, this study not only validates the effectiveness of the U-Net architecture for medical image segmentation but also underscores its practical utility in real-world clinical applications. Experimental evaluations reveal that integrating a 3 × 3 convolutional kernel within the spatial attention (SA) module results in superior segmentation performance on T1-weighted hippocampal MRI images. Remarkably, the highest recorded IoU, Dice scores, and accuracy reached 0.9345, 0.9512, and 0.98, respectively, on the dataset [34], while on the dataset [35], the model achieved IoU scores of 0.9816/0.8132 (train/validation), Dice scores of 0.9907/0.8963 (train/validation), and accuracy of 0.9952/0.9832 (train/validation), substantially outperforming existing approaches and setting a new benchmark in hippocampal segmentation accuracy for both the [34,35] datasets. While the ADNI-derived hippocampus dataset provides high-quality annotations, its small size may limit generalization and increase overfitting risks for complex 3D architectures. The single-source origin may also reduce robustness to inter-scanner variability. To address these limitations, we validated our model on the Medical Segmentation Decathlon hippocampus dataset, where it demonstrated consistent performance improvements, confirming broader clinical generalizability. Statistical validation via paired t-tests (*p* < 0.05) and 5-fold cross-validation confirms significant improvements over baselines, ensuring robust and reliable performance.

This study enhances the conventional UNet architecture by integrating dual attention mechanisms and optimizing convolutional kernel sizes within the spatial attention module. These modifications substantially improve the model’s ability to capture the complex spatial features of the hippocampal region, which is crucial due to its structural variability in brain MRI scans. Precise hippocampal segmentation is critical for early detection and monitoring of neurodegenerative disorders such as Alzheimer’s disease. The proposed deep learning framework advances automated hippocampal volumetric assessment and incorporating AI-driven segmentation into clinical decision-support systems can improve diagnostic accuracy and operational efficiency.

Future work will focus on enhancing clinical translational value through volumetric consistency analysis, hippocampal subfield segmentation, and correlation studies with cognitive assessments to bridge the gap between technical accuracy and clinical utility.

## Figures and Tables

**Table 1:** Confusion matrix for assessing model performance.

Predicted value	True value		
		Negative	Positive
	Negative	True Negative (TN)	True Positive (TP)
	Positive	False Negative (FN)	False Positive (FP)

**Table 2:** Environment configuration used for model development and training.

Component	Specification
Operating System	Windows 11 Professional Edition
Processor (CPU)	Intel^®^ Core ™ i7 – 12700 CPU @ 2.30 GHz
Memory (RAM)	40 GB
System Architecture	64-bit operating system, x64 processor
Graphics Card (GPU)	NVIDIA GeForce RTX 3080Ti (12 GB VRAM)

**Table 3:** The datasets used to test hippocampus segmentation in our experiment.

Category	ADNI Dataset Details	Decathlon Dataset Details
Dataset Modality	T1-weighted MRI	T1-weighted MRI
Total patients	135	390
Slices per patient	189	30–34
Slices with Masks	30	9–10
Patients Train/Test set	100/35	260/130
Images Train/Test set	18,900/6615	9270/4499
Total MRI images	25,515	13,769

**Table 4:** Training results based on different convolutional block configurations of the SA module. Bold highlights the highest values in each column.

Convolutional Block	Precision	Recall	Dice Coeff	IOU
SA module not added	0.8752	**0.9618**	0.8723	0.8548
1 × 1	0.8543	0.9605	0.8512	0.8335
3 × 3	**0.8960**	0.9607	**0.8916**	**0.8739**
5 × 5	0.8929	0.9588	0.8882	0.8701
7 × 7	0.8639	0.9601	0.8595	0.8415

**Table 5:** Training results under ablation experiments without using image enhancement methods. Bold highlights the highest values in each column.

Models	Precision	Recall	Dice Coeff	IOU
U-Net	0.7191	**0.9629**	0.7188	0.7008
U-Net + SA	0.8835	0.9608	0.8797	0.8619
U-Net + ISA	0.7265	0.9624	0.7257	0.7077
CSDA-UNet	**0.8862**	0.9604	**0.8820**	**0.8641**

**Table 6:** Training results under ablation experiments using image enhancement. Bold highlights the highest values in each column.

Dataset	Models	Precision	Recall	F1 Score	Dice Coeff	IOU
ADNI	U-Net	0.9439	**0.9587**	0.9302	0.9365	0.9193
ADNI	U-Net + SA	0.9549	0.9580	0.9307	0.9464	0.9291
ADNI	U-Net + ISA	0.9487	0.9552	0.9391	0.9381	0.9206
ADNI	CSDA-UNet	**0.9644**	0.9553	**0.9529**	**0.9512**	**0.9345**
Decathlon	CSDA-UNet	-	-	**0.9907/0.8963**	**0.9907/0.8963**	**0.9816/0.8132**

**Table 7:** Statistical Comparison of Dice Coefficient and IoU for Hippocampal Segmentation Models on ADNI and Decathlon Datasets. Bold highlights the highest values in each column.

Dataset	Models	Dice Coeff	Dice 95% CI	Dice *p*-Value	IOU	IoU 95% CI	IoU *p*-Value
ADNI	U-Net	0.9365	[0.932, 0.941]	-	0.9193	[0.914, 0.924]	-
ADNI	U-Net + SA	0.9464	[0.942, 0.951]	0.012	0.9291	[0.925, 0.933]	0.015
ADNI	U-Net + ISA	0.9381	[0.934, 0.942]	0.623	0.9206	[0.916, 0.925]	0541
ADNI	CSDA-UNet	**0.9512**	**[0.947, 0.955]**	**0.003**	**0.9345**	**0.930, 0.939**	**0.004**
Decathlon	CSDA-UNet	**0.9907/0.8963**	**[0.987, 0.994]/[0.890, 0.902]**	**-**	**0.9816/0.8132**	**[0.978, 0.985]/[0.807, 0.819**	**-**

**Table 8:** Comparison of the proposed method with other approaches using the same dataset. Bold highlights the highest values in each column.

Method	Dataset	Model	Dice Coeff	Accuracy
Nisha et al., 2022 [36]	ADNI	SSGAN	0.85	0.95
Das et al., 2022 [37]	ADNI	Modified 3D-UNet	0.9343	0.90
Lei Yang et al., 2023 [38]	Decathlon	Morphological ViT	0.90–0.886	-
Xiao Zhiyong et al., 2024 [39]	Decathlon	Swin-UNETR	0.878	-
Proposed CSDA-UNet	ADNI	CSDA-UNet	**0.9512**	**0.98**
Proposed CSDA-UNet	Decathlon	CSDA-UNet	**0.9907/0.8963**	**0.9952/0.9832**

## Data Availability

The original contributions presented in this study are included in the article. Further inquiries can be directed to the corresponding author.
